# Shexiang Baoxin Pill Alleviates the Atherosclerotic Lesions in Mice via Improving Inflammation Response and Inhibiting Lipid Accumulation in the Arterial Wall

**DOI:** 10.1155/2019/6710759

**Published:** 2019-07-11

**Authors:** Li Lu, Yating Qin, Xinxin Zhang, Chen Chen, Xiangyu Xu, Chingwen Yu, Xiaomei Guo

**Affiliations:** Department of Cardiology, Tongji Hospital, Tongji Medical College, Huazhong University of Science and Technology, Wuhan 430030, China

## Abstract

Epidemiological studies have demonstrated that cardiovascular diseases (CVDs) are the leading cause of death in the world. Atherosclerosis, a kind of chronic vascular disorder related to multiple pathogenic processes, has been reported to be an underlying cause of CVDs. Shexiang Baoxin Pill (SBP) is a traditional Chinese medicine formulation and has been broadly used for the treatment of CVDs in East Asia. However, whether SBP affects the development of atherosclerosis is poorly understood. The aim of this study was to investigate the antiatherosclerotic roles and relevant mechanisms of SBP in apolipoprotein E knockout mice. Our results showed that SBP treatment markedly decreased the size of atherosclerotic plaques of the entire aorta and the aortic sinus. Biochemical analyses indicated that SBP gavage improved oxidative stress in vivo, as seen by the level elevation of SOD, CAT, and GSH and the level reduction of MDA, H_2_O_2_, and MPO. Moreover, the concentration of MCP-1, IFN-*γ*, and IL-17A was reduced, and the content of IL-10 and TGF-*β*1 was increased in the serum from SBP-treated mice. We discovered that the expression levels of inflammatory factors including VCAM-1, ICAM-1, IL-6, and IL-2 in the vascular wall of the SBP group were also decreased in comparison with those of the normal saline group. Moreover, we found that SBP alleviated the activation of inflammation-related pathways in the aorta tissue, as seen by the level elevation of Mfn2 and reduced phosphorylation of p38, JNK, and NF-*κ*B. Furthermore, western blot showed that SBP administration reduced the level of SR-A and LOX-1 and elevated the content of LXR*α*, ABCA1, and ABCG1 in the arterial wall, indicating that SBP was capable of alleviating lipid influx and facilitating lipid efflux. In conclusion, our data suggested that SBP exerted antiatherosclerotic effects via improving inflammation response and inhibiting lipid accumulation.

## 1. Introduction

Although rapid advancements in medical care have been seen in the past years, cardiovascular diseases (CVDs) remain the main health problem in the world, resulting in more than 30% of all deaths per year [1]. Atherosclerosis, a chronic vascular disorder characterized by the formation of lipid-laden plaques in the tunica intima, has been demonstrated to be an underlying cause of CVDs, such as coronary artery diseases (CADs) [2]. It has been indicated that the arterial inflammation is deeply involved in the initiation and development of atherosclerotic lesions [3, 4]. When exposed to atherogenic factors, such as excess reactive oxidative species (ROS) and proinflammatory cytokines, vascular endothelial cells in a “quiescent situation” switch into an “activated state” and produce abundant adhesion molecules and chemokines, contributing to blood monocytes binding to the surface of the artery wall and infiltrating into the subendothelium. Then, monocytes differentiate into macrophages which produce cytokines to aggravate vascular inflammation and promote atherosclerosis development [5–8]. In addition, macrophages sequestered in the subendothelium ingest plenty of lipids and then transform into foam cells which are pivotal components of atherosclerotic lesions [9]. Several studies indicate that the lipid influx and efflux of the vascular wall are regulated by the scavenger receptors (SRs) and reverse cholesterol transporters (RCTs), respectively. In the atherogenic environment, the level increase of SRs and content decrease of RCTs could facilitate foam cell formation and atherosclerosis progression [10, 11]. Thus, it is effective to attenuate the development of plaque lesions through decreasing proinflammatory factors and regulating the expression of SRs and RCTs.

**Figure 1:**
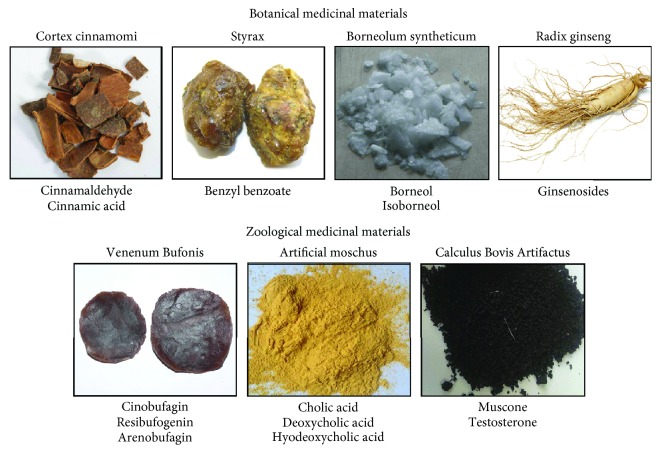
The medicinal materials contained in SBP. According to Chinese Pharmacopoeia 2015, SBP consists of seven medicinal materials. The upper part presents the botanical medicinal materials and corresponding bioactive extracts, and the below part shows the zoological medicinal materials and relevant bioactive extracts in SBP, separately.

Shexiang Baoxin Pill (SBP) is known as a traditional Chinese medicine formulation and has been widely used in Asian countries to treat diseases and improve health. It has been reported that SBP consists of seven medicinal materials: artificial moschus, radix ginseng, cortex cinnamomi, Styrax, Venenum Bufonis, Calculus Bovis Artifactus, and borneolum syntheticum (Figure 1) [12]. There are multiple studies demonstrating that bioactive extractives from the components of SBP including muscone, ginsenoside, cinnamaldehyde, and borneol could provide protective effects for the cardiovascular system via promoting angiogenesis, regulating cellular apoptosis, ameliorating lipid disposition, and improving oxidative stress [13–16]. In addition, SBP has been indicated to reduce the level of inflammatory cytokines in the circulation of metabolic syndrome rats and hyperlipidemic rabbits [17, 18]. As the progression of atherosclerosis is associated with multiple pathological factors including inflammatory response, oxidative stress, and lipid accumulation [19], SBP is likely to alleviate the development of atherosclerosis by improving these pathogenic processes. Moreover, in terms of clinical practice, SBP has been proved to display important roles in treating CADs which are mainly caused by atherosclerosis [12, 20]. Thus, SBP might be an effective agent inhibiting the progression of atherosclerosis. However, there are rare researches exploring the roles of SBP in the development of atheroma lesions until now. In this study, we investigated whether SBP administration exerted beneficial roles in attenuating the progression of atherosclerotic plaques and then analyzed relevant molecular mechanisms in apolipoprotein E-deficient (apoE^−/−^) mice.

## 2. Materials and Methods

### 2.1. Drugs

SBP (Chinese Pharmacopoeia 2015, approval number Z31020068) used in this study was offered by Shanghai Hutchison Pharmaceuticals (batch number 191523, Shanghai, China).

### 2.2. Animal Experiment

The present study process was conducted according to the National Institutes of Health Guide for the Care and Use of Laboratory Animals and was approved by the Institutional Animal Care and Use Committee of Tongji Medical College, Huazhong University Science and Technology, Wuhan, China (approval number TJ-A20170602).

8-week-old apoE^−/−^ mice with the C57BL/6 background were provided by Vital River Laboratory Animal Technology Co. Ltd. (Beijing, China) and were maintained in an air conditioned room (22 ± 2°C). All animals were kept under a 12 h light/12 h dark cycle with free access to food and water and were acclimatized to the environment prior to the initiation of experiment. Then, apoE^−/−^ mice at 10 weeks old were randomly divided into two groups. The mice were fed with high-fat diet (HFD, 21% fat and 0.15% cholesterol) supplemented with oral gavage of SBP (25 mg/kg/day) or normal saline (NS) for 20 weeks (*n* = 12 each group). The dosage of SBP was evaluated based on previously published animal studies and clinical guidelines [17, 21]. The physiological parameters including body weight, abdominal perimeter, food intake, blood pressure, and number of pulses were monitored during the study. At the end of the experiment, all animals were fasted overnight and were sacrificed. Then, the blood samples, heart tissues, and aortic tissues were collected for further study.

### 2.3. Assessment of Atherosclerotic Lesions in the Entire Aorta

En face lesions stained with Oil Red O (ORO, St. Louis, USA) in the entire aorta were quantified to analyze the range of atherosclerosis as previously reported. Briefly, the adipose and connective tissues which adhered to the tunica adventitia were rapidly removed under a stereo microscope. The whole aorta from the arch to the iliac bifurcation was carefully excised, opened longitudinally, pinned flat, and fixed in 4% paraformaldehyde solution overnight. Then, the aorta was washed three times with distilled water and was stained with 0.5% ORO working solution out of light for 2 h at room temperature. Subsequently, the aorta was immersed into 70% ethanol for destaining and then was rinsed with distilled water. The images were captured by a digital camera, and the atheroma plaques were stained in red color. The extent of aortic atherosclerosis was evaluated as the percentage of ORO-positive stained red area in relation to the area of the entire aorta luminal surface and was quantified using the Image-pro plus 6.0 software (Media Cybernetics, USA). The measurement of plaque lesion area was performed by members irrelevant to the study processes.

### 2.4. Morphometric and Histological Analysis

The mouse heart and connective aorta tissues were dissected and put in 4% paraformaldehyde overnight. Then, the tissues were embedded in optimum cutting temperature compound and were frozen at -80°C for the next step. Cryosections of the aortic sinus at 8 *μ*m thickness were made with a cryostat, and the transverse sections were then stained with ORO for the measurement of lipid deposition in the aortic sinus.

Paraffin sections of the aortic sinus were produced according to previous protocols. In short, the mouse heart and aorta tissues were excised and fixed in 4% paraformaldehyde for 24 h, followed by rinse and dehydration. Then, the tissues were embedded in the paraffin. Afterwards, the paraffin slicing machine was applied for cross sectioning, and 5 *μ*m thick sections were obtained. Then, Hematoxylin-Eosin (HE, Beyotime Biotechnology, Beijing, China) staining of the aortic sinus paraffin section was performed for detecting the size of atherosclerotic lesions. Moreover, cross-sections were stained with the antibody specific for CD68 (ABclonal, Boston, USA) for the immunohistochemistry analysis.

### 2.5. Determination of Serum Biochemical Parameters

The serum samples were gathered by centrifugation of blood samples at 3000 rpm for 10 min at 4°C and then were kept at -80°C. The concentrations of serum superoxide dismutase (SOD), catalase (CAT), reduced glutathione (GSH), malondialdehyde (MDA), hydrogen peroxide (H_2_O_2_), and myeloperoxidase (MPO) were measured by kits from Nanjing Jiancheng Bioengineering Institute in accordance with the manufacturer's protocols.

### 2.6. Detection of Circulating Cytokines

After the mouse serum was separated, the contents of the serum MCP-1, IFN-*γ*, IL-10 (Boster Biological Technology, China), IL-17A, and TGF-*β*1 (NeoBioscience, China) were detected by the enzyme-linked immunosorbent assay (ELISA) kits following the manufacturer's instructions.

### 2.7. Western Blot

The expression of proteins in the aorta tissue was analyzed by western blot. Briefly, the protein extracts were acquired by lysing the aorta tissues with the lysis buffer containing 1% protease inhibitors and centrifuging at 4°C for 15 min at 12000 g. Then, a BCA kit (Boster Biological Technology, China) was used to quantify the concentration of isolated proteins. An equal amount of protein (20 *μ*g) for each sample was subjected to SDS-PAGE (10% gel) and then was transferred to a PVDF membrane. The membrane was blocked with TBST containing 5% nonfat milk and then was incubated overnight at 4°C with specific primary antibodies including liver X receptor *α* (LXR*α*), ATP-binding membrane cassette transport protein (ABC) A1, macrophage scavenger receptor A (SR-A), lectin-like ox-LDL receptor 1 (LOX-1), intercellular adhesion molecule 1 (ICAM-1), and vascular cell adhesion molecule 1 (VCAM-1) (Abcam, Cambridge, UK); peroxisome proliferator-activated receptor *γ* (PPAR*γ*) (Santa Cruz Biotechnology, Texas, USA); mitofusin 2 (Mfn2), NF-*κ*B, p-NF-*κ*B, ERK1/2, p-ERK1/2, p38, p-p38, JNK, and p-JNK (Cell Signaling Technology, Boston, USA); CD36, ABCG1, CD68, and GAPDH (ABclonal, Boston, USA); and IL-6 and IL-2 (Affinity Biosciences, OH, USA). Subsequently, the membrane was rinsed with TBST for three times and was exposed to suitable secondary HRP-conjugated antibodies. The protein on the membrane was detected by the ECL kit, and band intensities were quantified by the ImageJ software (NIH, USA).

### 2.8. Quantitative Reverse Transcription Polymerase Chain Reaction (qRT-PCR)

The total RNA was extracted from the aortic tissues using Trizol reagent (Takara, Japan) in accordance with the manufacturer's protocols. Total RNA (1 *μ*g) was then converted to cDNA by the RT kits (Takara, Japan). qRT-PCR was performed on a Roche LightCycler480 System (Roche, USA) using a TB Green™ Premix Ex Taq™ II kit (Takara, Japan) to measure and analyze the expression of specific genes including IL-6, IL-2, VCAM-1, ICAM-1, LXR*α*, ABCA1, and ABCG1. The primer sequences (Sangon Biotech, China) used in this study were listed in Table 1. The amplification steps were as follows: denaturing at 95°C for 30 s, followed by 40 cycles of PCR reaction at 95°C for 5 s and 60°C for 20 s, then with a final dissociation stage at 95°C for 5 s, 60°C for 1 min, and 95°C for 0 s. The level of GAPDH was used as an internal control.

### 2.9. Statistical Analysis

The data in this study was expressed as the mean ± standard deviation (SD). The significant differences between two groups were assessed using Student's unpaired *t*-test. Data analysis was conducted by the SPSS version 21.0 software (IBM, Chicago, USA). The statistical significance was defined as *p* < 0.05.

## 3. Results

### 3.1. The Influence of SBP on the Physiological Parameters of Mice

To investigate the effects of SBP on the physiological characteristics of mice, parameters including body weight, abdominal perimeter, energy intake, blood pressure, and number of pulse were measured during the period of the experiment. As shown in Table 2, the differences in weight gain, abdominal girth, calorie intake, systolic blood pressure, and pulse rate between the two groups were not significant at each stage of the study, indicating that SBP had no roles in affecting the physiological characteristics of mice.

### 3.2. SBP Administration Significantly Alleviated the Development of Atherosclerosis

**Figure 2:**
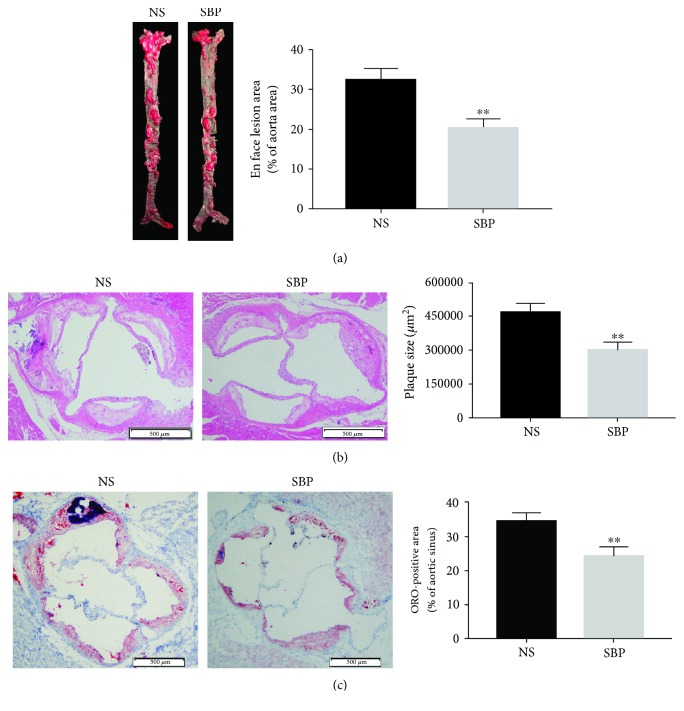
The effects of SBP on the development of atherosclerosis. (a) The lesion area of the entire aorta was detected by ORO staining. (b) The size of atheroma plaques in the aortic sinus was measured by HE staining. (c) Cryosections were stained with ORO to analyze the lipid disposition in the area of aortic sinus. The data was expressed as the mean ± SD, *n* = 6 for each group. ^∗∗^*p* < 0.01 vs. the NS group.

It is reported that apoE^−/−^ mice fed by HFD have been widely used for establishing the animal model of atherosclerosis [22, 23]. At first, we investigated the effects of SBP on atherosclerosis through measuring the size of plaque lesions in the entire aorta of the apoE^−/−^ mice fed by HFD. We found that the size of plaque lesions of the whole aorta was markedly decreased in the group of SBP gavage when compared to that in the group of NS gavage (Figure 2(a)). As the burden of atheroma plaques in the aortic sinus was another important indicator used for evaluating the severity of atherosclerosis, we then analyzed HE-stained paraffin sections of the aortic sinus and found that SBP treatment obviously reduced the size of atherosclerotic lesions in the aortic sinus (Figure 2(b)). In addition, the results of ORO-stained cryosections revealed that the size of lipid deposition in the aortic sinus from the SBP-administrated mice was markedly reduced when compared to that from the NS-treated mice (Figure 2(c)).

### 3.3. SBP Administration Improved the Antioxidative Abilities in the Circulation

**Figure 3:**
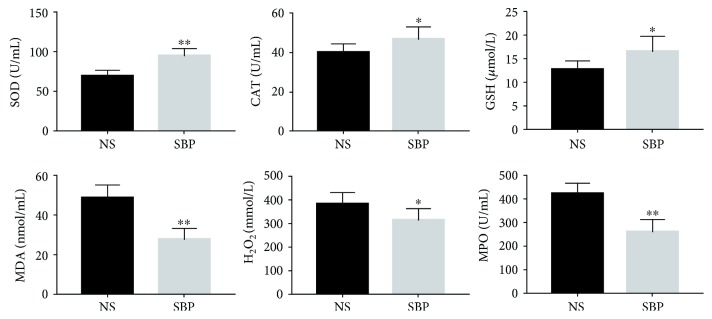
The levels of indicators reflecting the antioxidative abilities and the oxidative injury in the circulation of apoE^−/−^ mice. Results were expressed as mean ± SD. ^∗^*p* < 0.05, ^∗∗^*p* < 0.01 vs. the NS group, *n* = 7 per group.

It is well accepted that ROS-induced oxidative stress is capable of facilitating the progression of atherosclerosis by activating the inflammation-related pathways in arterial walls [6]. Then, we investigated whether SBP treatment enhanced the antioxidative abilities in vivo. As shown in Figure 3, the levels of antioxidants including SOD, CAT, and GSH were found to be increased in the circulation of SBP-treated mice. Moreover, MDA, H_2_O_2_, and MPO, which were positively correlated with the oxidative injury, showed reduced contents in the serum of the SBP group. These results suggested that SBP was capable of enhancing antioxidative abilities and improving the oxidative damage in the circulation of apoE^−/−^ mice.

### 3.4. SBP Regulated the Levels of Serum Cytokines

**Figure 4:**
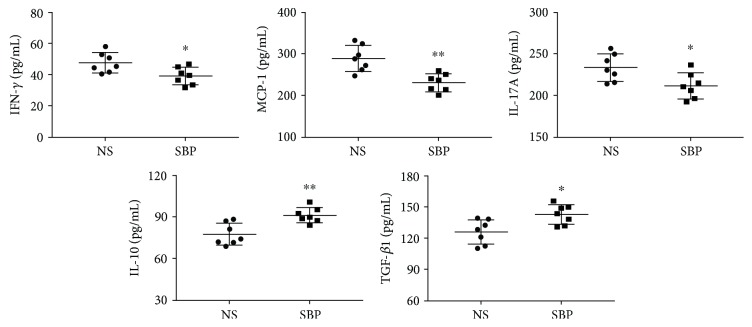
The effects of SBP treatment on the expression of inflammatory cytokines in the circulation. Levels of serum IFN-*γ*, MCP-1, IL-17A, IL10, and TGF-*β*1 were measured using ELISA kits. The values represented the mean ± SD, *n* = 7 per group. ^∗^*p* < 0.05, ^∗∗^*p* < 0.01 vs. the NS group.

There is evidence suggesting that a variety of inflammation-related cytokines in the circulation is associated with the development of atherosclerosis [24]. For determining whether the antiatherosclerotic roles of SBP could be explained by the alleviation of inflammation, we measured the levels of circulating cytokines using ELISA kits and discovered that SBP significantly decreased the concentrations of proinflammatory factors including MCP-1, IFN-*γ*, and IL-17A. Meanwhile, the levels of IL-10 and TGF-*β*1 which were regarded as important anti-inflammatory agents were showed to be elevated in the serum of the SBP group when compared to that in the serum of the NS group (Figure 4).

### 3.5. SBP Suppressed the Inflammatory Responses in the Aorta of apoE^−/−^ Mice

**Figure 5:**
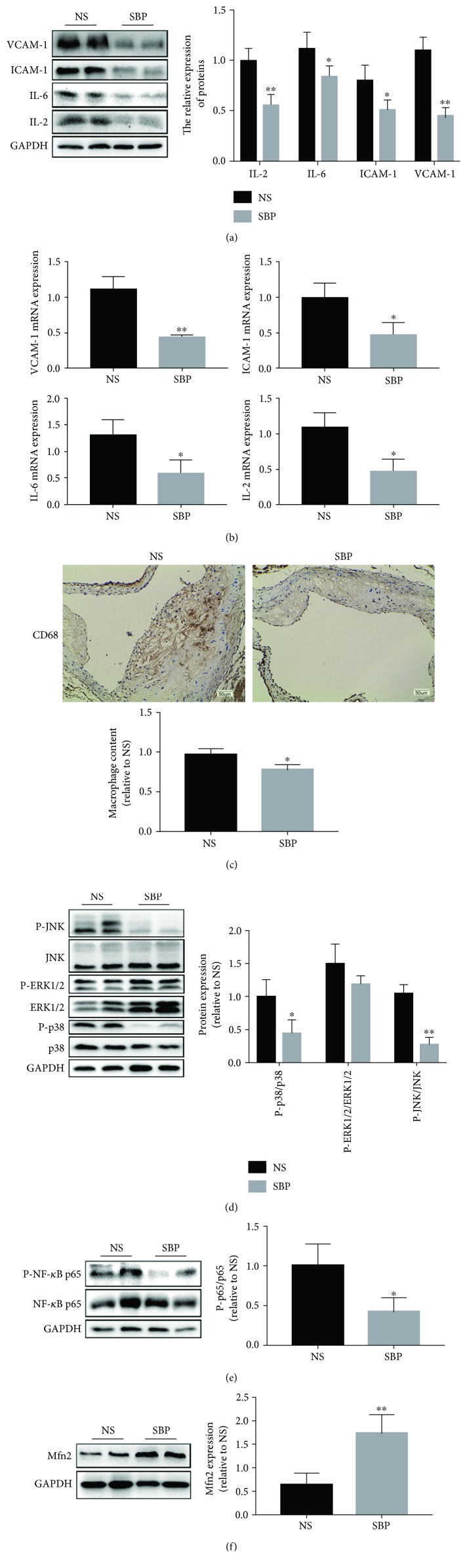
The inhibitory roles of SBP in vascular inflammation. (a) The expression of inflammatory proteins was quantified by western blot. (b) The mRNA expression of VCAM-1, ICAM-1, IL-6, and IL-2 was measured by qRT-PCR. (c) The content of macrophages in the lesion area of aortic sinus was investigated by immunohistochemical analysis for CD68 which was a hallmark of macrophages. (d-f) Signal molecules involved in the inflammation-related pathways were detected with western blot. The results were presented as the mean ± SD, ^∗^*p* < 0.05, ^∗∗^*p* < 0.01 vs. the NS group.

Given that the vascular inflammation was demonstrated to be a pivotal pathological process involved in the initiation and progression of atherosclerosis [25, 26], we detected inflammation-related molecules in the aortic tissues. As shown in Figures 5(a) and 5(b), SBP administration decreased the expressions of proinflammatory factors including VCAM-1, ICAM-1, IL-6, and IL-2 both at the transcriptional and translational level in the arterial tissues, which further substantiated the inflammation-resolving abilities of SBP. In addition, the immunohistochemical result of CD68 staining showed that SBP treatment reduced the content of macrophages in the vascular wall (Figure 5(c)), which might be ascribed to the SBP-induced expression decrease of VCAM-1 and ICAM-1, considering that VCAM-1 and ICAM-1 were crucial adhesion molecules inducing macrophage accumulation into the subendothelium. Then, to explain the results of SBP-triggered relief of the vascular wall inflammation, signaling proteins associated with inflammatory cascades were measured. Our data indicated that the activities of NF-*κ*B and mitogen-activated protein kinases (MAPKs) which were transcriptional regulators mediating inflammation factor generation were reduced in the vascular wall of the SBP group, as seen by the phosphorylated level decrease of NF-*κ*B p65, JNK, and p38. But the difference in the phosphorylated level of ERK1/2 between the two groups was not significant (Figures 5(d) and 5(e)). In addition, Mfn2, acting as a mediator inhibiting the activities of NF-*κ*B and MAPKs, was also found to be upregulated in the SBP group when compared to that in the NS group (Figure 5(f)). These results suggested that SBP-induced attenuation of inflammation might be a vital component of the antiatherosclerotic mechanisms of SBP.

### 3.6. SBP Mediated the Level of Factors Implicated in Lipid Disposition in the Aortic Wall

**Figure 6:**
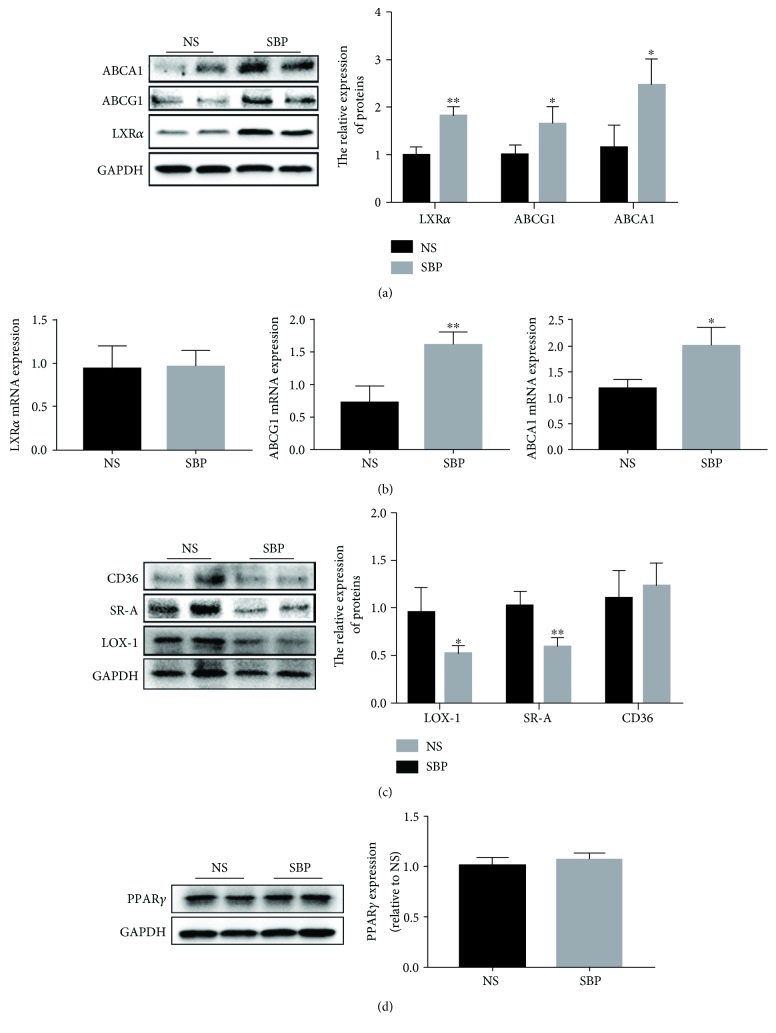
SBP regulated the expression of bioactive factors responsible for lipid disposition in the vascular wall. (a) The molecules associated with lipid efflux from the aorta were detected by western blot. (b) qRT-PCR was used to determine the mRNA levels of LXR*α*, ABCG1, and ABCA1. (c) The proteins facilitating the influx of lipid were measured by western blot. (d) The protein expression level of PPAR*γ* in the aortic tissues. The data was expressed as the mean ± SD, *n* = 4 per group. ^∗^*p* < 0.05, ^∗∗^*p* < 0.01 vs. the NS group.

When the lipid uptake capacity of the aortic tissues is enhanced or the ability of lipid efflux is impaired, excessive lipids accumulate into the arterial wall, contributing to the aggravation of the vascular inflammation and formation of foam cells, thereby facilitating the progression of atherosclerosis [11]. Then, to investigate whether SBP exerted antiatherosclerotic roles by inhibiting lipid disposition in the vascular wall, we detected the expression levels of SRs and RCTs which were responsible for lipid uptake and excretion separately. Western blot analysis indicated that the protein content of LXR*α*, ABCA1, and ABCG1 was increased in the SBP group in comparison with that in the NS group (Figure 6(a)). Moreover, the results of qRT-PCR showed that SBP treatment also elevated the expression of ABCA1 and ABCG1 at the transcriptional level, but the difference in the mRNA level of LXR*α* between the two groups was not significant (Figure 6(b)). In addition, we found that there was a level decline of SR-A and LOX-1 in the aorta of SBP-treated mice (Figure 6(c)). Nonetheless, the content of another SR CD36 and its upstream regulator PPAR*γ* was not affected by SBP administration (Figures 6(c) and 6(d)).

## 4. Discussion

With the characteristics of the highest death rate, atherosclerosis-related CVDs are the leading threat to public health worldwide [2, 27]. Previous studies have demonstrated that atherosclerosis is featured by a series of complex pathophysiological processes containing inflammatory response and subendothelial lipid accumulation [19]. SBP has been broadly used in Asia for the treatment of CVDs in the past decades. In this study, we discovered that oral gavage of SBP markedly alleviated the development of atherosclerosis in apoE^−/−^ mice. Afterwards, we found that the antiatherosclerotic mechanisms of SBP were attributed to the amelioration of inflammation reaction and suppression of lipid disposition in the vascular wall.

The findings from Ross and other studies have suggested the contributing roles of inflammation in the initiation and progression of atherosclerotic lesions [3, 4, 28]. It is clarified that circulating proinflammatory factors trigger the malfunction of the arterial endothelial barrier, leading to blood leukocyte mobilization into the subendothelial space, thereby facilitating the damage of the vascular structure and expansion of atheroma lesions [19, 24, 29]. Thus, we evaluated the anti-inflammatory effects of SBP by detecting the levels of inflammation-related cytokines in the circulation of apoE^−/−^mice. MCP-1, as an efficient chemotactic factor to monocytes, is involved in the vascular inflammation, and the absence of MCP-1 is found to mitigate atherosclerosis progression [30]. IFN-*γ* and IL-17A are reported to be capable of inducing macrophages to generate vast proinflammatory substances. Highly expression levels of IFN-*γ* and IL-17A are shown in the animal model of atherosclerosis [31, 32], while IL-10 and TGF-*β*1 have been proven to act as key anti-inflammatory agents in vivo and content elevation of IL-10 and TGF-*β*1 effectively attenuate the expansion of atheroma plaques [33, 34]. Results in this study indicated that treatment with SBP obviously reduced the level of serum MCP-1, IFN-*γ*, and IL-17A and increased the level of IL-10 and TGF-*β*1, respectively, demonstrating SBP's anti-inflammatory abilities. Furthermore, several lines of evidences illustrate that atherogenic effects of oxidative stress could be partly explained by ROS-induced inflammation responses in the vascular intima [35, 36]. Our data delineated that SBP administration elevated the contents of antioxidants and improved oxidative stress in the bloodstream of apoE^−/−^ mice. In addition, Mfn2, a mitochondrial membrane protein capable of improving mitochondrial dysfunction and alleviating oxidative stress [37], was found to be upregulated in the SBP group, which suggested that SBP-induced attenuation of oxidative injury might be partly associated with the level increase of Mfn2. These findings suggested that protective roles of SBP against the progression of atherosclerosis were associated with the decrease of proinflammatory stimuli and increase of anti-inflammatory factors in the circulation.

**Figure 7:**
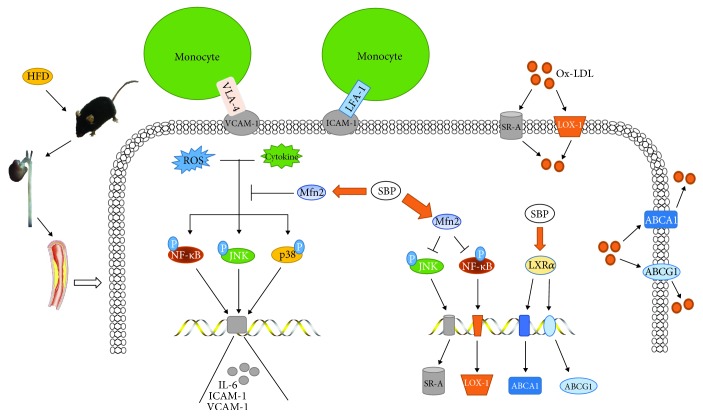
Schematic description of the protective mechanisms by which SBP attenuated the development of atherosclerosis in apoE^−/−^ mice fed by HFD. ROS and proinflammatory cytokines activated the signaling molecules including NF-*κ*B, JNK, and p38 to promote the production of inflammation factors. SBP treatment increased the level of Mfn2 and then suppressed the activities of NF-*κ*B, JNK, and p38, thereby reducing the generation of inflammatory cytokines and monocyte accumulation. Meanwhile, SBP elevated the contents of reverse cholesterol transporters and reduced the levels of scavenger receptors via regulating the signal transduction of relevant pathways in the vascular wall, which reduced the lipid influx and accelerated the efflux, leading to repression of foam cell formation.

Since the arterial intima directly contacts the bloodstream, circulating proatherosclerotic substances are prone to induce the expression of inflammatory adhesion molecules in the vascular intima, such as VCAM-1 and ICAM-1, facilitating firm attachment of monocytes to the vessel lining [7, 8]. Then, the adherent monocytes migrate into the subendothelial area and differentiate into macrophages which produce proinflammatory factors like IL-6, thereby aggravating the inflammation reaction in the aorta and accelerating the progression of atherosclerotic plaques [19]. Our findings showed that the levels of ICAM-1, VCAM-1, and IL-6 and the amount of macrophages were reduced in the arterial wall from the SBP group, suggesting that SBP also played beneficial roles in inhibiting inflammatory responses occurred in the aorta. Moreover, we discovered that SBP administration obviously decreased the expression of IL-2 in the aortic tissues. Although the precise roles of IL-2 in atherosclerosis are not well clarified, substantial research evidences have demonstrated the potent roles of IL-2 in stimulating lymphocytes to produce inflammatory cytokines, such as IL-6, TNF-*α*, and IFN-*γ* [7, 24]. Thus, the protective roles of SBP against vascular inflammation also might be associated with the expression reduction of IL-2. Among the inflammation-related signal molecules, NF-*κ*B is a pivotal upper stream regulator responsible for mediating the expression of multiple proinflammatory proteins including interleukins and adhesion molecules which participate in the atherosclerosis development [24, 38]. Furthermore, MAPKs including JNK, p38, and ERK1/2 have been reported to play roles in the regulation of inflammation expansion by triggering the transcription of relevant genes [39, 40]. Mfn2 is reported to possess anti-inflammatory properties and is demonstrated to suppress the signal transductions involving NF-*κ*B and MAPK pathways [37, 41, 42]. In this study, the increased expression of Mfn2 and reduced activities of JNK, p38, and NF-*κ*B were observed in the SBP group, suggesting that oral administration of SBP interrupted the signal flow of proinflammatory pathways in the aorta. However, the transduction of ERK1/2 signal cascade was not affected by SBP treatment. Since it was indicated that there were agents regulating apoptotic processes through inhibiting the ERK1/2 signal pathway but activating p38 and JNK cascade [43], in this study, the activity of the ERK1/2 pathway might also be different from other pathways. Moreover, our previous data and several researches had proved the regulatory roles of Mfn2-mediated inhibition of the ERK1/2 pathway in cellular proliferative and apoptotic processes, but not in the inflammatory process [42, 44, 45]. Therefore, these findings suggested that the pathways responsible for SBP-induced inflammation inhibition in the present study might not involve the Mfn2/ERK1/2 pathway and remained to be further investigated. Collectively, these results indicated that anti-inflammatory effects of SBP might be explained by elevation of the Mfn2 expression and then suppression of JNK, p38, and NF-*κ*B pathway transduction, accompanied by reduction of cytokine biosynthesis and leukocyte accumulation (Figure 7).

Cumulative evidences from cellular and animal experiments have demonstrated that lipid disposition in the vascular wall acts as an essential pathogenic event at each stage of atherosclerosis [9]. Depending on scavenger receptor-mediated endocytosis, multiple cells including macrophages and vascular smooth muscle cells in the aortic wall internalize plentiful lipoproteins in the subendothelial layer, leading to the formation of foam cells which are crucial components of atherosclerotic lesions. SR-A, CD36, and LOX-1 are the main SRs controlling the majority of lipid uptake [10, 11]. It is reported that the level increase of CD36 and LOX-1 is positively correlated with the severity of atheroma plaques and agents reducing the expression of LOX-1 and SR-A effectively inhibit the progression of atherosclerotic lesions [46–48]. Our findings showed that SBP significantly decreased the content of SR-A and LOX-1 but did not reduce the expression of CD36 in the aorta of apoE^−/−^ mice. A possible reason accounting for this situation was that SBP regulated the expression of these receptors by mediating different signal cascades. Briefly, SR-A and LOX-1 were found to be the downstream molecules of the NF-*κ*B cascade and the JNK pathway separately, both of which were suppressed by SBP in the present study [47, 48]. Nevertheless, CD36 was found to be regulated by signaling molecule PPAR*γ* which presented a similar expression level between the two groups [49]. Reverse cholesterol transport is a physiological process associated with lipid migration from peripheral tissues like the vascular wall back to the liver for final excretion. As a vital defense process against the development of atherosclerosis, reverse cholesterol transport contains some steps and the first one is the lipid efflux from cells within the arterial wall, which is modulated by membrane transporters including ABCA1 and ABCG1 [10, 11]. Then, we observed that SBP treatment triggered the protein level increase of ABCA1 and ABCG1 as well as the upstream transcriptional regulator LXR*α* in aortic tissues. In addition, qRT-PCR analysis showed that SBP treatment effectively elevated the mRNA level of ABCA1 and ABCG1 but did not induce the increase of LXR*α* mRNA, indicating that SBP facilitated the process of reverse cholesterol transport via enhancing the translational activity of LXR*α* mRNA, followed by the expression upregulation of downstream effector ABCA1 and ABCG1 both at the transcriptional and translational level. These above results indicated that improvement of pathway activities regarding lipid influx and efflux and subsequently suppression of foam cell formation might be implicated in the antiatherosclerotic abilities of SBP (Figure 7).

## 5. Conclusions

Taken together, the present study shows that oral administration with SBP potently reduces the lesion size of atherosclerosis in apoE^−/−^ mice induced by HFD. Our results provide the first evidence that SBP displays antiatherosclerotic roles that involve the decrease of circulating proinflammatory factors, improvement of vascular inflammation, and inhibition of lipid disposition in the arterial wall, which indicates that SBP has great potentials to serve as an effective agent for the treatment of atherosclerosis in clinical application.

## Figures and Tables

**Table 1:** The sequences of primers used for qRT-PCR in this study.

Gene name	Primer forward sequence (5′-3′)	Primer reverse sequence (5′-3′)
IL-6	GGAGCCCACCAAGAACGATAG	GTGAAGTAGGGAAGGCCGTG
IL-2	AGATGAACTTGGACCTCTGCG	GAAAGTCCACCACAGTTGCTG
VCAM-1	CTGGGAAGCTGGAACGAAGT	GCCAAACACTTGACCGTGAC
ICAM-1	CCGTGGGGAGGAGATACTGA	TCGAGCTTTGGGATGGTAGC
LXR*α*	AGTGTCATCAAGGGAGCACG	TCGACACTCCTGGCATTTCC
ABCA1	GTTAGGAAACCTGCTGCCCT	CGGGAGAAGAGCGTGCTAAT
ABCG1	CCTGCCTCCTCTTCTACCCT	TGCCTTGGGTTTGGGTTTCT
GAPDH	TGTGAACGGATTTGGCCGTA	GATGGGCTTCCCGTTGATGA

**Table 2:** The physiological parameters of mice during the experiment (*n* = 12 per group).

	Intervention at week 0		Intervention at week 10		Intervention at week 20	
	NS	SBP	NS	SBP	NS	SBP
Body weight (g)	25.2 ± 1.57	25.08 ± 1.34	31.07 ± 3.18	30.48 ± 3.11	36.4 ± 3.36	34.55 ± 4.01
Abdominal girth (cm)	7.11 ± 0.29	7.06 ± 0.3	8.08 ± 0.52	8.12 ± 0.53	8.58 ± 0.46	8.49 ± 0.42
Calorie intake (kcal)	71.26 ± 5.51	69.78 ± 3.72	104.1 ± 9.98	108.6 ± 9.01	107.4 ± 12.43	101.7 ± 10.58
Systolic blood pressure^∗^ (mmHg)	113.6 ± 4.76	115.6 ± 6.8	119.2 ± 6.92	116.4 ± 6.78	115.8 ± 7.37	114.4 ± 7.22
Pulse rate^∗^ (beats per minute)	567.8 ± 18.08	576.1 ± 21.45	582.6 ± 20.22	591.8 ± 24.7	565.2 ± 21.1	570.8 ± 20.26

^∗^The systolic blood pressure and pulse numbers of the mouse tail artery were acquired using a computerized and noninvasive tail-cuff system.

## Data Availability

The data used to support the findings of this study are available from the corresponding author upon request.
